# Aptamer‐targeted DNA nanostructures with doxorubicin to treat protein tyrosine kinase 7‐positive tumours

**DOI:** 10.1111/cpr.12511

**Published:** 2018-10-12

**Authors:** Mengting Liu, Wenjuan Ma, Qianshun Li, Dan Zhao, Xiaoru Shao, Qian Huang, Liying Hao, Yunfeng Lin

**Affiliations:** ^1^ State Key Laboratory of Oral Diseases National Clinical Research Center for Oral Diseases, West China Hospital of Stomatology, Sichuan University Chengdu China

## Abstract

**Objectives:**

Aptamer sgc8c is a short DNA sequence that can target protein tyrosine kinase 7 (PTK7), which was overexpressed on many tumour cells. This study aimed to fabricate a novelty DNA nanostructure drug delivery system target on PTK7‐positive cells—CCRF‐CEM (human T‐cell ALL).

**Methods:**

Aptamer‐modified tetrahedron DNA was synthesized through one‐step thermal annealing process. The sgc8c‐TDNs (s‐TDNs) loading DOX complexes were applied to investigate the effect to PTK7‐negative and ‐positive cells.

**Results:**

When s‐TDN:DOX acted on PTK7‐positive and ‐negative cells respectively, the complexes exhibited specific toxic effect on PTK7‐positive cells but not on PTK7‐negative Ramos cells in vitro research.

**Conclusions:**

In this work, we successfully constructed a PTK7‐targeting aptamer‐guided DNA tetrahedral nanostructure (s‐TDN) as a drug delivery system via a facile one‐pot synthesis method. The results showed that s‐TDN:DOX exhibited enhanced cytotoxicity against PTK7‐positive CCRF‐CEM cells, with a minor effect against PTK7‐negative Ramos cells. Hence, this functionalized TDNs drug delivery system displayed its potential application in targeting PTK7‐positive tumour T‐cell acute lymphoblastic leukaemia.

## 1 INTRODUCTION

According to the reports of Leukemia and Lymphoma Society, 62 103 people were diagnosed with leukaemia in 2017^1^ and the initial cost of care for each leukaemia patient is almost $36 000 (from NIH).^2^ The average life expectancy is severely decreased for acute lymphoblastic leukaemia (ALL), as the age group below 9 years is at higher risk of this disease.^3^ Until now, doxorubicin, as a widely used anticarcinogen, is the most frequently used treatment to ALL. However, owing to its nonspecific delivery and multiple serious side effects such as cardiotoxicity, alopecia, myelosuppression, and leucocytosis have limited its application.^4^ Therefore, an effective drug delivery system that targets therapeutics to leukaemia cells while minimizing toxicity to other cells is desirable.

Nanomaterial drug delivery system is a promising technology that offers advantages such as tumour‐specific accumulation of the drug, improvement in drug half‐life, and enhancement of drug bioavailability.^5^ Yet, the commonly used nanoscale carriers such as carbon tubes,^6^ lipid nanoparticles,^7^ and cationic polymers^8^ show suboptimal performance for their poor biocompatibilities.^9^ Tetrahedron DNA (TDNs), based on the canonical Watson‐Crick base pairing, with its inherent biocompatibility exhibits a stable structure, multiple decorated sites, mechanical rigidity, and homogeneous size and composition and is shown to display excellent characteristics for drug delivery.^9^, ^10^, ^11^ Previous studies^12^, ^13^, ^14^ have revealed TDNs could enter cells without any auxiliary agents, suggestive of its potential as an ideal delivery system.

Aptamers, also known as “chemical antibodies,” are small RNA/DNA sequences that are capable of binding to target entities ranging from small molecules to proteins.^15^, ^16^, ^17^ In comparison with traditional antibodies, aptamers offer multiple advantages such as high stability,^18^ better compatibility,^19^ versatile chemical modification,^20^ and quick chemical production,^21^ making them attractive candidates for targeted cancer therapy. Sgc8c is a DNA sequence with 42 nucleotides that is known to specifically bind to protein tyrosine kinase 7 (PTK‐7), a cell membrane protein known to be overexpressed on CCRF‐CEM (human T‐cell ALL) cells^22^, ^23^ and many other tumours^24^ such as colon and gastric cancer. Given its potential application as a targeted aptamer, sgc8c may be used to fabricate a drug delivery system, namely, sgc8c‐TDN (s‐TDN) to selectively target ALL cells.

**Figure 1:**
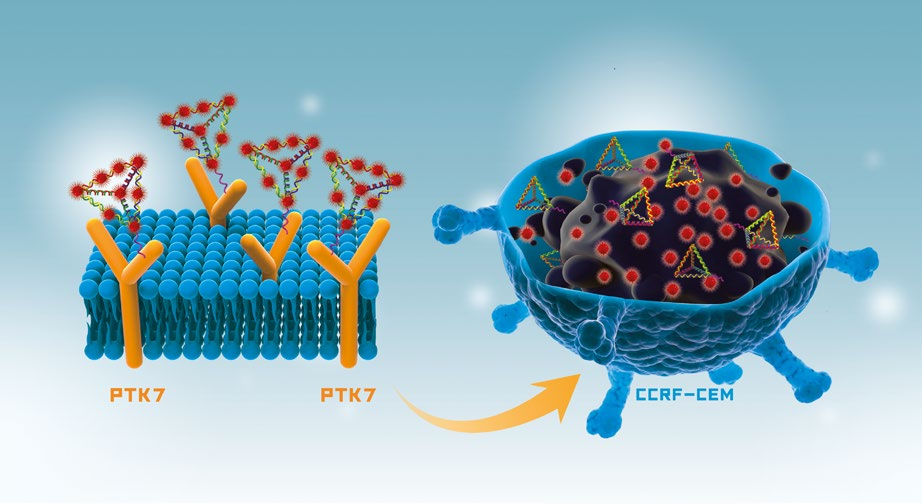
Schematic illustration of the aptamer‐modified DNA tetrahedron for the selective delivery of doxorubicin in PTK7‐positive CCRF‐CEM cells

Considering that DOX has the ability to intercalate into ‐GC‐rich regions of DNA,^25^, ^26^, ^27^ we fabricated s‐TDN:DOX complexes as a targeted drug delivery system (Figure 1). Furthermore, we evaluated the behaviour of this nanosystem in vitro. Laser scanning confocal microscopy (LSCM) and flow cytometry (FCM) experiments were performed to investigate the cellular uptake of s‐TDNs. In addition, the cellular uptake of DOX was also evaluated by FCM. Cell cytotoxicity tests revealed that s‐TDN:DOX was more cytotoxic to target cells as compared with untargeted cells. We conclude that sgc8c‐TDN is a feasible targeted drug delivery system and that s‐TDN:DOX may serve as an ideal targeted treatment against ALL or other PTK7‐positive tumours.

## 2 MATERIALS AND METHODS

### 2.1 Cell culture

We obtained PTK7‐positive cells (CCRF‐CEM, human T‐cell ALL) and PTK7‐negative cell (Ramos, human Burkitt's lymphoma) from Cell Center of Chinese Academy of Medical Sciences (Beijing, China). Cells were cultured in RPMI‐1640 medium supplemented with 10% (v/v) FBS (Gibco, CA, USA) and 1% (v/v) penicillin‐streptomycin solution (HyClone, Pittsburgh, PA, USA) under a standard humidified atmosphere of 5% CO_2_ at 37°C.^28^, ^29^, ^30^

### 2.2 Preparation of TDN and aptamer‐modified TDN

**Figure 2:**
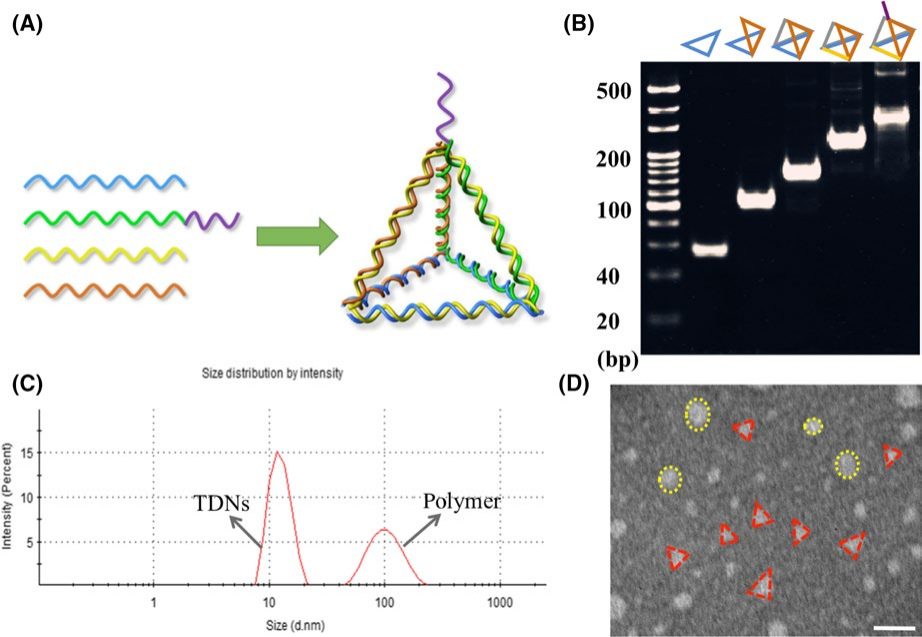
Characterization of TDN and Apt‐TDN. (A) Schematic illustration of s‐TDN synthesis. (B) Eight per cent polyacrylamide gel electrophoresis (PAGE) for confirmation. (C) TEM images of s‐TDNs. Scale bars are 20 nm. (D) Hydrodynamic size of TDNs measured by dynamic light scattering

As reported in our previous study,^31^, ^32^, ^33^ TDNs were synthesized by four single‐stranded DNA (synthesized and purified by TaKaRa, Dalian, China) molecules S1‐4 (Table 1) through one‐step thermal annealing process in TM buffer (10 mmol/L Tris‐HCl [pH 8.0] and 50 mmol/L magnesium chloride [MgCl_2_]). Each single‐stranded DNA molecule was used in equimolar concentration and formed one triangle of TDNs (Figure 2A). In the TDN, every edge was formed via highly specific canonical Watson‐Crick base pairing by two different single‐stranded DNAs.^34^, ^35^, ^36^ Aptamer‐modified TDNs were fabricated under same conditions as TDNs except that S2 was replaced by S5, which is an S2 extended with the aptamer sgc8c.

**Table 1:** The specific sequence of each single‐stranded DNA

ssDNA	Direction	Detail sequence
S1	5′→3′	ATTTATCACCCGCCATAGTAGACGTATCACCA
		GGCAGTTGAGACGAACATTCCTAAGTCTGAA
S2	5′→3′	ACATGCGAGGGTCCAATACCGACGATTACAGC
		TTGCTACACGATTCAGACTTAGGAATGTTCG
S3	5′→3′	ACTACTATGGCGGGTGATAAAACGTGTAGCAA
		GCTGTAATCGACGGGAAGAGCATGCCCATCC
S4	5′→3′	ACGGTATTGGACCCTCGCATGACTCAACTGC
		CTGGTGATACGAGGATGGGCATGCTCTTCCCG
S5	5′→3′	ATCTAACTGCTGCGCCGCCGGGAAAATACTGTA
		CGGTTAGATTTTTACATGCGAGGGTCCAATACCG
		ACGATTACAGCTTGCTACACGATTCAGACTTAGG
		AATGTTCG

### 2.3 Characterization of TDNs and Apt‐TDNs

To confirm the successful synthesis of TDNs and Apt‐TDNs, molecular weights of DNA nanostructures were evaluated using 8% polyacrylamide gel electrophoresis. Transmission electronic microscopy (TEM; HT770, Hitachi, Tokyo, Japan) was performed to observe the morphology of s‐TDN. The size of TDN was evaluated by dynamic light scattering (DLS; Malvern Instruments Ltd, Malvern, UK).

### 2.4 Cellular uptake of TDNs and aptamer‐modified TDNs

To investigate the cellular uptake of the nanomaterial, single‐stranded DNA S1 was fluorescently tagged with cyanine‐5 (Cy5) fluorophore (red fluorescence). FCM was used to quantify the internalization of DNA nanostructure. CCRF‐CEM and Ramos cells were seeded in six‐well plates at an initial density of 5 × 10^4^ cells/mL without FBS. After 1 hour, regular culture medium (as negative control) and Cy5‐TDN and Cy5‐Apt‐TDN solutions (final concentration of 100 nmol/L for both) were added in the medium. The cells were incubated for 12 hours, followed by their collection and centrifugation at 200 ***g*** with three washes of phosphate‐buffered saline (PBS). The cells were suspended in 400 μL of PBS for FCM analysis. A total 15 000 cells were randomly collected and their fluorescence intensities were evaluated by a flow cytometer (FC500 Beckman, IL, USA). In addition, LSCM (FV500‐IX81; Olympus America Inc., Melville, NY, USA) was used to observe TDNs localization and uptake quality. These samples were cultured under the same condition as FCM test. At predetermined time point, cells were collected and fixed with 4% formaldehyde for 15 minutes at 4°C. After stained at room temperature with 10 μg/mL 4′,6′‐diamidino‐2‐phenylindole (DAPI) for 10 minutes, images were obtained by LSCM subsequently. In the process of the experiment, cells were washed thrice with PBS at every step and protected from light at all time.

### 2.5 Loading of DOX on TDNs and Apt‐TDNs

As the fluorescence of DOX would be quenched after its embedment into the double‐helix structure,^37^ the drug‐loading capacity of the DNA nanostructure could be evaluated by measuring the fluorescence intensity of DOX. In this study, different volumes of DOX (200 μmol/L) were mixed with TDNs and s‐TDNs in 96‐well plates to obtain different concentration ratios (1:5, 1:10, 1:20, 1:30, 1:40, 1:50, 1:60, 1:80, 1:100, and 1:150). After incubation at room temperature for 2 hours, the fluorescence spectra were measured with a microplate reader (VariOskan Flash 3001; Thermo, MA, USA) at wavelengths from 490 to 700 nm. All operations were carried out away from the light.

### 2.6 Cellular uptake of DOX

From the results of DOX‐loading capacity test, we concluded that 1:20 was optimal concentration ratio of TDNs and DOX for the encapsulation of all DOX molecules into the TDNs. Therefore, DOX was added into the DNA nanostructure solution (100 nmol/L) at a final concentration of 2 μmol/L and incubated at room temperature for 2 hours, resulting in the assembly of DOX‐loaded DNA nanostructure. Cells were treated with 100 μmol/L of nanocompounds mixed with serum‐free medium for 2 hours and samples were collected as prescribed for FCM analysis. The fluorescence of DOX in cells was evaluated by FCM.

### 2.7 Cell counting kit‐8 assay

To investigate the targeted toxicity of nanomaterials, cell counting kit‐8 (CCK‐8) assay was performed for the evaluation of the killing efficiency. Before the experiment, TDNs:DOX and s‐TDNs:DOX were synthesized at concentrations of 100 nmol/L DNA nanostructure and 2 μmol/L DOX. Cells (1 × 10^4^ cells/well) were cultured in serum‐free medium with free DOX, TDN:DOX, and s‐TDN:DOX at 37°C in an atmosphere of 5% CO_2_ for 2 hours. Following incubation, 75% of medium was replaced with fresh growth medium (10% FBS) and cells were incubated for another 48 hours. Subsequently, fresh serum‐free medium containing 10% (v%) of cell titre reagent (Dojindo Laboratory, Tokyo, Japan) was added to each well and the plates were incubated for 1 hour at 37°C for the evaluation of cytotoxicity. A microplate reader was used to record the absorbance value at 450 nm wavelength.

### 2.8 Statistical analysis

In this study, all tests were conducted in triplicate and verified repeatedly. One‐way analysis of variance (ANOV; SPSS 19.0: IBM Corp., Armonk, NY, USA) was employed to analyse the difference between treatment groups and controls, and Student's *t*‐test was applied to analyse the means of each pair of group. A value of *P *<* *0.05 was considered statistically significant all the time.

## 3 RESULTS

### 3.1 Synthesis and characterization of the DNA nanostructure

Here, a novel targeted DNA nanostructure as a drug delivery system was successfully prepared. As shown in Figure 2A, TDNs and s‐TDNs comprised four single‐stranded DNA molecules and each strand formed a triangle of the tetrahedron structure. Every side of the tetrahedron was composed of two sections of DNA strands displaying tight Watson‐Crick base pairing. As each single strand is about 60 bp (in Table 1), so we can calculate that TDNs is about 240 bp. Moreover, due to the modified with aptamer sgc8c, s‐TDNs ought to be about 280 bp. As shown in Figure 2B, the positions of TDNs and s‐TDNs matched well with the theoretical values of their molecular weights.^10^, ^33^ In addition, DLS was conducted to determine the size of TDNs. Two peaks were displayed on Figure 2C, where in the first peak corresponded to TDNs with a size of about 12 nm, consistent with the results of previous study,^13^, ^31^ and the second peak reflected the presence of some large polymers can also be formed during the synthesis process. As reported before, the formation of polymers is a common phenomenon during the reaction process.^10^ In addition, the morphology of TDNs was verified by TEM. Displayed on Figure 2D, several triangular nanostructures were observed along with circular structures of 100 nm, which corresponded to the polymers of TDNs in Figure 2C. Together these results suggest that TDNs and s‐TDNs were successfully prefabricated.

### 3.2 Aptamer modification facilitates TDN entry into target cells

**Figure 3:**
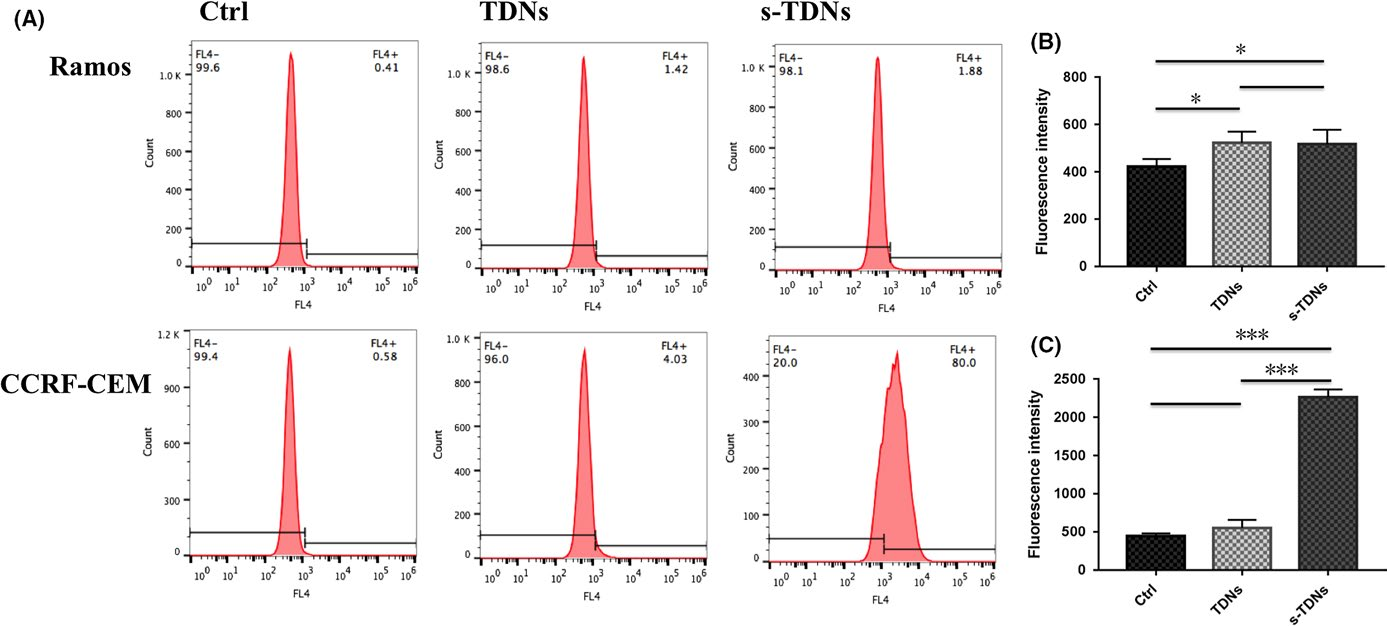
Flow cytometry analysis for the evaluation of the cellular uptake of TDN and aptamer‐modified TDN (100 nmol/L) after 12 h coculture. (A) Positive rate of cell uptake for cy5‐TDNs and cy‐5s‐TDNs. Mean fluorescence intensity for Ramos (B) and CCRF‐CEM (C). Statistical analysis: *P <* *0.05, **P <* *0.01, ***P <* *0.001

**Figure 4:**
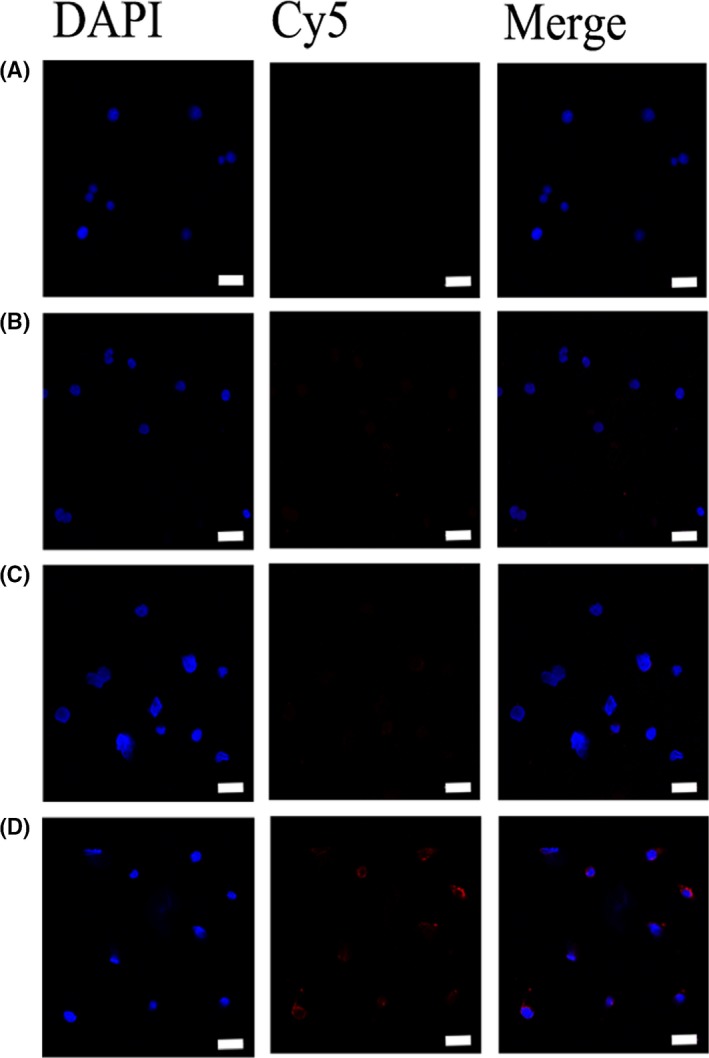
Laser confocal microscopy micrographs of Ramos (PTK7‐negative cell) and CCRF‐CEM (PTK7‐positive cell) incubated with TDN and s‐TDN (100 nmol/L) after 12 h (nucleus: blue; Cy5: red). (A) TDNs incubated with Ramos. (B) s‐TDNs incubated with Ramos. (C) TDNs incubated with CCRF‐CEM. (D) s‐TDNs incubated with CCRF‐CEM. Scale bars are 20 nm

To study the targeting effect of the aptamer, we treated CCRF‐CEM and Ramos cells with cy5‐TDNs and cy5‐s‐TDNs. After 12 hours of incubation, cells were collected and analysed by FCM. We found that the uptake efficiency of TDNs was low into both CCRF‐CEM and Ramos cells. However, s‐TDNs showed higher binding to CCRF‐CEM, but not Ramos, suggesting that the aptamer sgc8c could target PTK7 and facilitate TDNs entry into the cell (Figure 3A,B). As shown in Figure 3C, CCRF‐CEM cells showed up to four times higher uptake than Ramos cells. To demonstrate the phenomenon intuitively, DAPI was performed to make nucleus blue to observe cy5‐TDNs and cy5‐s‐TDNs in these cells. As nucleus of lymphocyte occupies about 90% volume of the whole cell, we can see the red TDNs is closed to the blue nucleus in Figure 4D. Compared with other group, Cy5 fluorescence intensity for cy5‐s‐TDNs was much stronger in CCRF‐CEM cells (Figure 4A‐C). Thus, aptamer sgc8c could not only help TDNs entry into the cell but also specifically target on PTK7‐positive cells (CCRF‐CEM), indicative of its potential application for the drug delivery of PTK7‐positive tumours.

### 3.3 Drug‐loading capacity of TDNs and s‐TDNs

**Figure 5:**
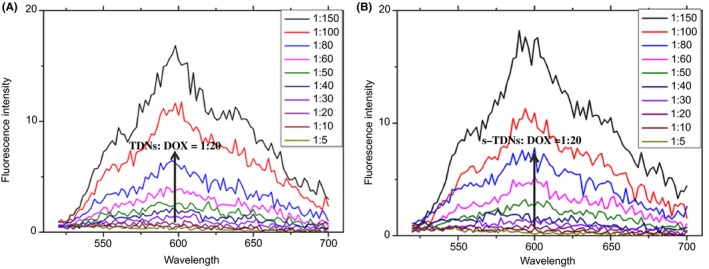
Drug‐loading capacities of TDNs and s‐TDNs. Fluorescence spectra of DOX solution mixed with TDNs (A) and s‐TDNs (B) at increasing molar ratios (concentration ratio of TDNs or s‐TDNs and DOX from top to bottom: 1:150, 1:100, 1:80, 1:60, 1:50, 1:40, 1:30, 1:20, 1:10, 1:5)

Drug‐loading capacity is one of the most important factors to evaluate the efficiency of the drug delivery system. Here, 100 nmol/L TDNs and s‐TDNs were individually mixed with DOX at increasing molar ratio for 1 hour at room temperature. As shown in Figure 5A, the fluorescence of DOX was completely quenched at TDNs to DOX molar ratio of 1:20, indicating each TDNs could carry 20 times DOX and revealing TDNs is an ideal drug vehicle. Interestingly, sgc8c‐decorated TDNs have not shown greater carrying capacity than TDNs (Figure 5B). It can be interpreted as sgc8c is an isolated single strand, which lacked the ability to bring DOX. TDNs and its derivative exhibit excellent characteristic as a drug delivery vehicle.

### 3.4 Maximum Dox uptake was observed in PTK7‐positive cells treated with s‐TDNs

**Figure 6:**
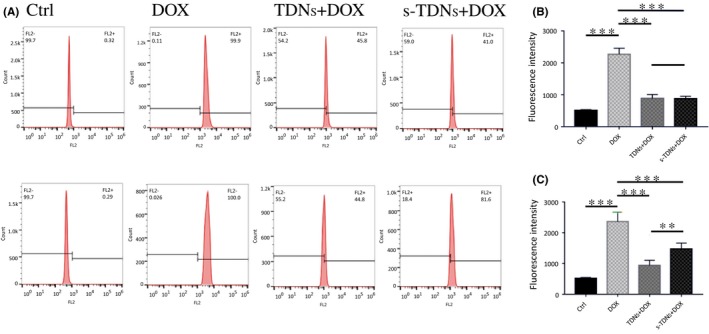
Cellular uptake of doxorubicin after 2 h coculture with free DOX, TDNs:DOX, and s‐TDNs:DOX. Flow cytometric analysis of the cells treated with free Dox, TDNs:Dox, and s‐TDNs:Dox for 2 h (A). The value of mean fluorescent intensity obtained from the cytometric analysis of Ramos (B) and CCRF‐CEM (C). Statistical analysis: *P <* *0.05, **P <* *0.01, ***P <* *0.001

Doxorubicin, a widely used antineoplastic drug, can be inserted into the DNA double‐stranded structure for the existed of flat aromatic rings in its molecule^38^ and is known to inhibit DNA duplication and RNA synthesis.^39^ Therefore, the delivery of high concentration of DOX to target cells is essential for the effective killing effect. DOX has multiple side effects for its nonselective feature and hence it is meaningful to decrease its nontargeted cellular uptake. FCM results (Figure 6A) showed that fluorescent signals of DOX generated from TDNs and s‐TDNs were similar in PTK7‐negative cells (Ramos); however, PTK7‐positive cells treated with s‐TDN:DOX showed approximately two times higher signal than those treated with TDNs:DOX. The value of mean fluorescent intensity of DOX (Figure 6B,C) was calculated from the FCM data via Flowjo.7.6. We can find that s‐TDNs carry more DOX to the target CCRF‐CEM cell not into Ramos. Thus, s‐TDNs promoted efficient DOX uptake into targeted cells as compared to untargeted TDNs.

### 3.5 s‐TDN:DOX exhibited specific toxic effect in PTK7‐positive cells in vitro

**Figure 7:**
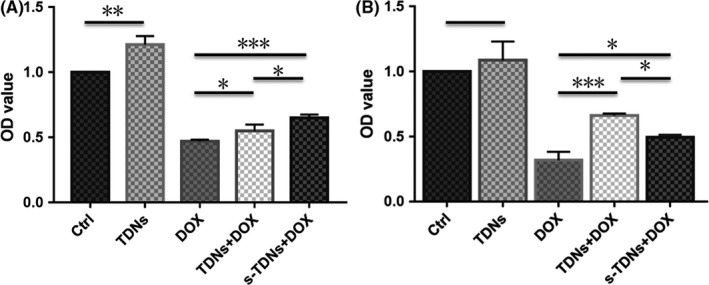
CCK‐8 assay of free DOX, TDNs:DOX, and s‐TDNs:DOX to PTK7‐negative cell (Ramos) and PTK7‐positive cell (CCRF‐CEM). (A) Cytotoxicity against Ramos cells treated with no agents (control), free DOX, TDN:DOX, and s‐TDN:DOX. (B) Cytotoxicity against CCRF‐CEM cells treated with no agents (control), free DOX, TDN:DOX, and s‐TDN:DOX. Statistical analysis: *P <* *0.05, **P <* *0.01, ***P <* *0.001

The results described above highlight the ability of the aptamer sgc8c to specifically target PTK7‐positive CCRF‐CEM cells. To evaluate whether s‐TDNs:DOX enhances the cytotoxicity in PTK7‐positive cells and decrease toxicity in PTK7‐negative cells, a CCK‐8 test was performed. As shown in Figure 7, free DOX showed similar and enhanced cytotoxicity against both PTK7‐positive and PTK7‐negative cells, which explained the reason for its side effects. On the other hand, TDNs:DOX and s‐TDNs:DOX showed no statistical difference in toxicity against PTK7‐negative cells (Figure 7A), which is in accord with DOX cellular uptake test that an equal amount of DOX was internalized in both cells. Surprisingly, with the aid of sgc8c, more TDNs that carry DOX are entering the CCRF‐CEM cells and resulted in greater toxicity in PTK7‐positive cells than in negative cells (Figure 7B). These results demonstrate that s‐TDNs:DOX could specifically inhibit the growth of PTK7‐positive CCRF‐CEM cells and reduce cytotoxicity against PTK7‐negative Ramos cells.

## 4 DISCUSSION

Leukaemia is one of the most common therioma and is known to seriously affect the health and lives of patients. Chemotherapy, often used as the priority treatment against leukaemia, has multiple side effects in the body, highlighting the need to develop a targeted delivery system.

In our present study, we found that the emerging DNA material TDNs could freely penetrate the cell plasma membrane without any transfection agent,^11^, ^40^, ^41^ suggestive of its potential as a promising drug carrier. The aptamer sgc8c in combination with nanocarriers such as carbon nanotubes^42^ and gold nanoparticles^43^ has been targeted to PTK7‐positive tumours and exhibited promising results in multiple studies; however, these nanocarriers showed more or less toxicity to cells; hence, there is an urgent need to construct a safe and targeted drug delivery system. Here, we fabricated a novel aptamer‐modified DNA nanostructure for the targeted delivery of DOX to PTK7‐positive cells CCRF‐CEM such that it minimizes DOX toxicity to untargeted cells.

We confirmed the ability of sgc8c to specifically target PTK7‐positive cells CCRF‐CEM to facilitate TDNs uptake using FCM and LSCM. As s‐TDNs are negatively charged nanomaterial, owing to electrostatic repulsion, it is unlikely that they were taken up via passive processes.^11^ In pervious study, Bagalkot et al^38^ revealed the uptake of aptamer A10 PSMA is by receptor‐mediated endocytic. As sgc8c has the same structure with A10 PSMA; therefore, we can infer that receptor‐mediated endocytic is the main mechanism of cellular uptake of s‐TDNs. Our in vitro study revealed that s‐TDN nanocarrier is a feasible vehicle for targeted drug delivery and that s‐TDN:DOX can kill target cells while reducing the cytotoxic effects against PTK7‐negative cells. TDNs are taken up by caveolin‐dependent pathway,^11^ which is a major kind of receptor‐mediated endocytic. And our results have demonstrated that sgc8c have ability to facilitate TDNs go into targeted cells. With continuously monitored the uptake process by confocal microscopy, a study^11^ found TDNs was transported to and trapped within the lysosome, which is an organelle that invading particles are degraded. As the degradation of TDNs, DOX were released from the TDNs subsequently, then diffused to the cytosol and finally to the nucleus to do its work. In conclusion, these findings suggest that the application of s‐TDN:DOX may be a potential strategy to curb PTK7‐positive tumours such as ALL under clinical settings.

The United States Food and Drug Administration has approved several aptamers for clinical use, including macugen for age‐related macular degeneration,^44^ as1411 for cancer treatment,^33^ and others.^45^ In our study, we successfully coupled TDNs to an aptamer, indicating the feasibility of constructing various apt‐TDNs as targeted drug delivery systems specific for particular organs and diseases, in the future.

## ACKNOWLEDGEMENT

This study was supported by the National Natural Science Foundation of China (81671031, 81470721) and Sichuan Province Youth Science and Technology Innovation Team (2014TD0001).
